# Occurrence of Body Posture Abnormalities in Overweight and Obese Children Aged 5–6 Years—Pilot Study

**DOI:** 10.3390/children11070849

**Published:** 2024-07-12

**Authors:** Alicja Bober, Aleksandra Kopaczyńska, Agnieszka Puk, Agnieszka Chwałczyńska

**Affiliations:** 1 Student Scientific Society, Wroclaw University of Health and Sport Sciences, 51-612 Wrocław, Poland; 2 Department of Human Biology, Wroclaw University of Health and Sport Sciences, 51-612 Wrocław, Poland

**Keywords:** posture in children, overweight in children, the impact of overweight on body posture, body composition and body posture

## Abstract

Objectives: The study aimed to assess the occurrence of body posture disorders and their changes under the influence of a physioprophylactic program in children depending on body weight. Methods: In the examined children, the general and segmental body composition and body posture were determined using a physiotherapeutic assessment based on the Kasperczyk method. Mass, overall, and segmental body composition were determined using the bioelectrical impedance method using a TANITA body composition analyzer. The study group of 76 children was divided due to body weight disorders into Group I (n = 51), in which BMI and fat mass values were within the normative limits for age and gender, and Group II (n = 19), comprising children whose body weight exceeded the norm and/or fat mass exceeded normative values. The examined children underwent a physioprophylactic. The program was conducted by qualified physiotherapists for 12 weeks, once a week for 30 min. The therapeutic program was focused on physioprevention of being overweight and the correction of body posture. Results: A distal distribution of fat mass was observed in the examined group. Asymmetry in the sagittal plane was found in 35% of children. No statistically significant differences were found in the presence of asymmetry in the sagittal plane between the groups. No statistically significant differences were found in the occurrence of posture irregularities between the groups. Conclusions: There were no changes in the weight and body posture of the examined children under the influence of the physiotherapy program. The lack of correlation in the examined group between body weight and posture irregularities in 5–6-year-olds may suggest the acquisition of posture defects as a consequence of the persistence of overweight or obesity.

## 1. Introduction

### 1.1. Body Posture in Preschool-Age Children

Body posture is one of the fundamental elements in the assessment of children. Despite being individualistic, numerous scales and tests have been developed to enable both objective and subjective evaluation. Proper body posture is defined as the alignment of individual body segments in a way that does not incur additional energy costs and does not cause discomfort or pain [1,2,3,4]. The silhouette’s shape changes throughout a child’s ontogenetic development until achieving structural maturity in adolescence [1,3]. The body posture of preschool-age children occurs during a period of slow growth, during which initial stabilization takes place. Continuous modifications in spinal curvature occur due to the torso’s growth and weak abdominal muscles. By the end of the preschool period, cervical lordosis stabilizes. Constant changes in silhouette alignment also occur in the chest and lower limbs. At the age of 6 years, the lower limbs straighten at the knee joints, and the child’s feet are properly arched both longitudinally and transversely [1,2,5].

### 1.2. Body Composition

The composition of the human body is influenced by various factors, including those independent of lifestyles, such as age, gender, genetic conditions, and ethnic origin, and those dependent on lifestyle, i.e., physical activity undertaken, diet, stress level. While changes can be made in some aspects, such as physical activity level or lifestyle, it is impossible to modify age and gender. Adhering to a healthy lifestyle is crucial, especially at a young age during development. The preschool period, compared to the previous developmental period, is characterized by a decrease in the growth rate of height and body weight. The child is growing, but there are no dynamic growth spurts during this period. At the age of 3–6, boys should grow approximately 15 cm, which corresponds to the average increase in body length during the first 12 months of life. The child’s body prepares for the initial period of posturogenesis, characterized by a significant increase in muscle mass due to bone thickening and muscular system development. The body posture of 5–6-year-olds becomes slenderer, with a reduction in fat mass, strengthening of abdominal muscles, and a balanced ratio of strength to body mass, allowing for a more correct posture [4,6,7,8,9,10,11,12,13].

### 1.3. The Impact of a Child’s Body Composition on Posture

During the preschool age, maintaining proper percentages of muscle tissue and fat mass are crucial to provide optimal conditions for development and trouble-free progression through stages. Excessive fat mass or its improper distribution can lead to functional and structural disorders in the developing body [10,14]. Such disparities can affect the spinal alignment, positioning of the lower limbs, and the arch of the feet—skeletal elements exposed to significant loading forces, with children of a young age lacking adequate stabilizing conditions to mitigate adverse effects. Excessive fat in the lower limbs may result in impaired loading mechanics. During this period, the physiological “fat pad” of the foot, which is responsible for flat feet, may alter the load on the feet, causing them to be excessively varus. At the same time, incorrect distribution of fat mass placed distally may cause reduced mobility of the lower limbs by increasing the circumference of the thighs, reducing muscle strength and, consequently, leading to the occurrence of knee valgus. Varus and valgus alignment of the knees disturbs the mechanics of foot loading, and if it persists for a longer period, it causes compensation at the level of the pelvis and lumbar spine [2,15,16,17].

The study aimed to assess the occurrence of body posture abnormalities depending on the body weight of children. Does the segmental distribution of fat and muscle mass correlate with the presence of posture irregularities? Will 12-week physioprophylactic classes change the weight and posture of preschool-age children?

## 2. Materials and Methods

The research project received approval from the Senate Committee on Research Ethics at Wroclaw University of Health and Sport Sciences (12/2019) and the study was conducted following the Declaration of Helsinki (registration number and name of trial registry ClinicalTrials.gov. NO. NCT 06419829).

Parents of all participants provided written informed consent before data collection began. Before starting the measurements and exercises, the child’s verbal consent to participate in the examination and exercises was obtained.

The research presented in this paper is part of a pilot project involving the assessment of the biological state of preschool and school children and the impact of targeted physical activity on body weight, posture, and physical fitness. One of the aims of this research was to test the methodology used in terms of its reproducibility on a group of preschool children.

Initial tests were carried out in January–February 2023, then a 12-week therapeutic program was carried out, and follow-up tests were carried out in May 2023.

The sample size was calculated using the G*Power 3.1.9.7 test by combining the difference between two independent means for a test power of 0.85 at a significance level of 0.05. The size of the study group should be 118 subjects. A total of 250 children who met the eligibility criteria for the group were invited to participate in the study. Of these, 124 took part in the project. Reasons for the reduction of the group were the lack of parents’ consent to participate in the project, absence from the kindergarten on the day of the examination, and the child’s lack of willingness to take part in the fitness test. Data from 78 people were qualified for analysis. The reason for exclusion from the analyzed group was low attendance at preschool classes during the research project (the child’s absence from classes was at least 50%).

### 2.1. Design

Children whose parents gave written consent to participate in the research and the child’s participation in a 12-week physiotherapy project qualified for the research project. Oral consent was obtained from children to participate in the study and therapeutic activities. The conditions for qualification to the study group were: no health contraindications to the use of the bioelectrical impedance method (body composition measurement), no medical contraindications to physical activity, and foot length exceeding 15 cm (technical condition for body weight measurement). The examined children’s body height was measured using a SECA 213 stadiometer. Weight, general, and segmental body composition were examined using an eight-electrode MC-780 body composition analyzer from TANITA. For the examined children, fat mass (FM% [%], FM [kg]), lean mass (FFM [kg]), and overall muscle mass (PMM), as well as segmental muscle mass for the right upper limb (RA), left upper limb (LA), right lower limb (RL), left lower limb (LL), and trunk (TR), were estimated [18,19,20]. Based on the obtained values of overall and segmental body composition, the fat-free mass index (FFF = FM/FFM) was calculated [7,8]. The Kasperczyk method was applied for the assessment of body posture, involving a physiotherapeutic evaluation in both standing and sitting positions. The evaluation included the symmetry of the head, shoulders, spinal curves, knee joints, and foot positioning. The subject took the assessment in sports clothes (shorts, T-shirt) without shoes and socks. Placed on a flat, rigid surface (wooden floor) in the resting position. The child remained in this position for about 1 min, then the joint was assessed. The symmetry of head position was examined—inclination in the sagittal plane (right–left), in the frontal plane (front–back), and complex movements (right-sided and left-sided rotation). The next level was the assessment of the position of the shoulder girdle—its symmetry by determining the position of the shoulders and the lower angles of the shoulder blades. The occurrence of lateral curvatures of the spine was assessed with the term “not present/present” (at which level, in which direction, whether compensation occurs at another level), and waist angles were also determined. The spine has three physiological curvatures as follows: lordosis, which is a natural forward bending of the spine in both the cervical and lumbar sections, and kyphosis, which is a natural curvature of the spine comprising an arcuate bending towards the back in the thoracic section. The conducted research assessed abnormalities in the physiological curvatures of the spine. The curvature of the spine in the frontal plane was assessed based on the spinous processes, their positioning in relation to the higher and lower sections, the cervical section (hyperlordosis, deepened lordosis, shallow lordosis, abolition of lordosis), thoracic section (flattened kyphosis, deepened kyphosis, abolition of kyphosis) and lumbar (flat lordosis, abolished lordosis, hyperlordosis). The lower limbs were assessed at the level of the hip girdle using the rear upper hip spines, knee position, and joint position.

The next assessment was performed each time on the right side. The front–back position of the head and the protrusion of the chin forward were assessed. The occurrence of shoulder pronation was assessed. The assessment of spine alignment in the frontal plane was completed. The tension of the abdominal wall was assessed. In the case of the lower limbs, the correctness of the pelvic position (anterior inclination, posterior inclination) was assessed using reference points, i.e., the posterior upper and anterior upper iliac spines, and the position of the knees (extension, hyperextension, flexion—without specifying degrees).

At the front, the head position, shoulder pronation, waist angles, pelvis position, and the knee joints were assessed. The foot position was assessed separately—correct/incorrect (varus, valgus, equinus–varus, flat) [8].

Body posture tests for all subjects were carried out by one person to eliminate measurement errors. The temperature in the room during the test was 23–25 °C. The entire examination for one child did not exceed 10 min.

### 2.2. Participants, Therapists, Centers

The study took place at preschool number 26 in Wrocław. After examining 200 children, 78 individuals qualified for the research group. The inclusion criteria for the research group were written consent from parents/legal guardians for their child to participate in the research project and the child’s age being 5–6 years. The selected children were divided into two groups based on abnormalities in body weight. Group I (NMC, n = 51) included children with normal body weight according to WHO standards, with BMI values for age and gender falling between the 5th and 85th percentiles, and with the percentage of body fat within normative ranges for age and gender (♀-15–25%; ♂-13–20%). Group II (PNMC, n = 27) consisted of children with above-normal body weight and/or a percentage of body fat exceeding 25% in girls and 20% in boys relative to total body weight [2,7,8,9,12,13,14,20,21].

### 2.3. Intervention

The examined children underwent a physioprophylactic program aimed at improving body weight and posture. The program was conducted by qualified physiotherapists for 12 weeks, once a week for 30 min. The exercise units were adapted to the age of the examined children (5 and 6 years old). The classes were carried out in groups of 10 people in the kindergarten gym, using equipment for posture correction therapy, sensory paths, and therapeutic sets for children.

The classes took place in the morning at least 45 min after breakfast and before the next meal. Each group had a specific time in terms of the day of the week and time of classes. All classes were conducted by the same team of therapists under the supervision of an experienced physiotherapist. At least three therapists were in the room with the children to correct the children during the exercises. Each class began with an introductory part—a 7-min warm-up, during which general fitness exercises were performed (marching, jogging, running, playing tag), and exercises for large joints (jumping jacks, upper limb swings in all planes, lower limb lunges). The main part included exercises strengthening the muscular corset and muscles of the lower and upper limbs. This part lasted from 15 to 18 min. The last part (up to 5 min) of each class included stretching exercises, calming down, and relaxation. After classes, the children returned to their kindergarten rooms.

### 2.4. Outcome Measures

Primary outcome: The expected result is a positive impact of the therapeutic program on the weight and body posture of preschool children.

Secondary outcome: Overweight and obesity at preschool age determine the occurrence of postural abnormalities in children

### 2.5. Data Analysis

Statistical analysis of the results was conducted using the Statistica 13.3 software. Descriptive statistics (mean, standard deviation, group size) were used. The normality of distribution was assessed using the Shapiro–Wilk test. Non-parametric statistics for dependent groups, the Wilcoxon test, were applied for intra-group comparisons (right side vs. left side). For inter-group comparisons, the U Mann–Whitney test for independent groups was used. The chi-square test was employed for quantitative comparisons. Spearman’s rank correlation was used to assess the relationship between body composition values and body posture. The statistical significance level was set at *p* < 0.05.

## 3. Results

The study compared both groups in terms of height, weight, and body structure. Children from the second group obtained higher values in all examined elements compared to the first group (Table 1).

Distant distribution of fat mass was observed. In both groups, the percentage of fat tissue is higher in the upper limbs than in the lower limbs, and it is higher on the left side of the body compared to the right side. The overall muscle mass of the upper and lower limbs differs statistically regardless of the group. In Group II, significantly higher percentage values of overall fat tissue were observed in all body segments compared to Group I (Table 2).

The analysis of the obtained values in the conducted assessment of body posture allowed for the identification of asymmetry in the sagittal plane of the body in 35% of the examined children, while 8% of the participants exhibited asymmetry in head positioning in the frontal plane. The assessment of spinal curves revealed the presence of hyperlordosis in the cervical spine in 18% of the examined children. In the lumbar spine, hyperlordosis was observed in 51% of individuals. Additionally, hyperkyphosis in the thoracic spine was identified in 18% of the participants. No statistically significant differences were found in the occurrence of posture irregularities based on the group (Table 3).

In the examined Group I, a negative, statistically significant mean correlation was observed between body weight, FFM, PMM values, and posture irregularities, as well as within the knee and foot areas. A similar correlation was noted between BMI and body posture and irregularities in the foot area. In Group II, a positive, statistically significant mean correlation was found between the occurrence of irregularities in the lumbar spine and the body weight of FFM and PMM. FM% showed a negative, statistically significant correlation with the occurrence of body posture asymmetry in Group II. When assessing the correlations between the structure of the lower limbs and the body posture of the examined children from Group I, a negative, statistically significant average correlation was observed between the values of the body composition of the lower limbs, FFM, and PMM on both the left and right sides, and the body posture of the subjects and the position of the lower limbs, knees and feet. In Group II, a positive average, statistically significant correlation was found between the position of the lumbar spine and the body composition of the right lower limb, FFM, and PMM. Additionally, FM% showed a negative, statistically significant correlation with the occurrence of body posture asymmetry in Group II. The correlation results are presented in Table 4.

After completing the therapeutic program, it was found to be imperfect and lacking results. The main problem in this age group during the period under study (February–May) was attendance at classes, which did not exceed 50% on average. Only 17% of the surveyed children participated in more than 75% of the classes. Such a low turnout resulted in no changes in body composition. The children grew slightly and increased their body weight, which was a physiological process, but they did not change the proportions of body composition. The program used was sub-threshold for this age group in terms of frequency and intensity of classes and did not result in improving body posture. A positive aspect of this program was the increased awareness of kindergarten workers who, participating in classes for children, asked about the correct positions that children should take during classes, rest and meals, what to pay attention to while playing, and what forms of physical activity should be permanently included in the class program to be implemented by preschool teachers.

## 4. Discussion

The occurrence of overweight in children in Poland remains a topic not fully explored. Numerous studies have been conducted, yet further exploration of the issue is necessary, as the number of children with overweight and increased fat mass continues to rise [2,3,5,6,8,15,16,17]. According to the Polish Society for Obesity Treatment, in Poland, overweight or obesity affects 12.2% of boys and 10% of girls in preschool-age children [21,22]. Meanwhile, the World Health Organization (WHO) reports that overweight and obesity are present in 32% of Polish children aged 7–9, ranking Poland 8th among the surveyed countries in Europe [21,22].

The results of the presented research and the research available in the literature are often contradictory. They lack unidirectionality. Such diversity of results, or rather directions of changes, indicates the need for further research and the development of uniform procedures and research methods.

### 4.1. Overweight and Spine Abnormalities

The content of fat mass influences the positioning of individual segments of a child’s body. According to Wyszyńska et al., this includes factors such as the inclination of the thoracolumbar spine, which affects the forward tilt of the entire torso. In the case of children with a high level of physical activity, the values of torso forward tilt were lower [5]. Based on the studies by Labecka et al., a correlation was found between the occurrence of overweight in children and the prevalence of angular values of thoracic kyphosis over lumbar lordosis [1]. However, in contrast to Wyszyńska et al.‘s research, Labecka et al. state that children with overweight exhibited a reduced angle of torso backward tilt [1,5]. Similar results in school-age children were obtained by Wilczyński et al., who observed abnormalities in body posture in more than half (58.92%) of the subjects, most often scoliotic posture and reduced kyphosis [23]. Based on the conducted studies in the group of children with above-normal body weight, we observed irregularities (hyperlordosis, loss of lordosis) in the lumbar spine. This indicates the presence of a prevalence of lumbar lordosis over thoracic kyphosis. This relationship allows us to conclude that in overweight children, the values of torso tilt are higher. The difference between our research results and Wilczyński’s results may be due to the age difference of the studied children (approximately 6 years of difference), the development period—preschool/school age (the beginning of puberty), and a different lifestyle—an active preschooler/a student sitting at a bench. More frequent discussion of the correct body posture in the peri-pubertal age is related to the relative stabilization of growth and skeletal structure in the spine [8,16,23,24,25,26].

### 4.2. Overweight and Abnormalities in the Lower Limbs

Based on the conducted studies by Maciałczyk-Paprocka et al., which included children aged 7–12 with overweight, posture irregularities were observed, most commonly including knock-knees and flat feet. The authors also highlight that existing disorders in the body predispose to the occurrence of posture defects in the later life of children [4]. In research conducted by Kolarova et al. on a group of preschoolers, abnormalities were found mainly in the feet. Using the Wolański method on a group of 6–8-year-olds, researchers found that over 50% of the respondents had flat feet requiring correction through corrective classes or appropriately selected insoles [27]. The analysis of the results of their research showed statistically significant correlations between the body posture abnormalities of the respondents and knee and foot disorders, and anthropometric values in the group of children with normal body weight. However, in the second group (children with above-normal body weight), a positive, statistically significant correlation was observed between anthropometric values and irregularities in the alignment of the lumbar spine segment. These results allow us to conclude that there is no connection between overweight and posture disorders in 5–6-year-old children. This may indicate that the preschool age is too early for such studies, as it is a period when many changes occur in the child’s body as elements of posture formation.

### 4.3. Overweight and General Body Posture Abnormalities

Maciałczyk-Paprocka et al., based on their conducted research, state that to prevent the development of posture defects in both children with normal body weight and those who are overweight, it is important to implement preventive measures [2]. Other researchers come to similar conclusions. Despite the lack of correlation between overweight and posture irregularities in children, the authors emphasize the need for gymnastic exercises as a preventive measure against the development of posture defects that may arise later as a consequence of weight disorders occurring at a young age [28]. The authors also highlight the importance of physical activity for children; based on their research, functional exercises and movements have a positive impact on improving the posture of overweight children [3]. Just like in the articles mentioned above, in our conducted research, there was no statistically significant correlation between the presence of posture irregularities in children and their above-normal body weight. However, this does not necessarily mean that disorders will not appear later as a result of persistent overweight. Therefore, adopting a healthy lifestyle, including a proper diet and regular physical activity, may have positive effects in the future. Araujo et al., examining a cohort of over 2000 children in Portugal, observed that for the assessment of body posture at the age of four and seven years, the child’s gender is a more important differentiating factor than body weight abnormalities. They found that the body mass indexes of fat and fat-free mass were weakly related to the lumbar angle [29]. Similar results of screening studies of Spanish children aged 6–12 years were presented by Grijon-Nogueron et al., emphasizing the importance of gender and not body weight on the occurrence of postural abnormalities assessed using the body posture index (FPI) method [30].

In the study by Lopez-Peralta et al., despite the lack of correlation between overweight and the presence of posture irregularities, the researchers also point out the presence of other disorders that are worth noting. Based on the research, lower bone density values in the lumbar spine were observed in children with increased values of fat mass [31]. The results presented by Lopez-Peralta et al. and many other authors indicate the need to assess not only body composition but also bone mineral density, which is negatively affected by overweight and obesity [31,32,33,34,35].

The correlation between the occurrence of overweight in children and irregularities in their posture remains an open issue. Despite the lack of statistically significant results confirming this association in the group of preschool children, the present disorders should not be underestimated, as they can negatively impact the lives of children in the future. Further diagnostics are necessary, along with adherence to preventive recommendations, which can reduce current irregularities and potentially have a positive impact on the posture of children in the future.

### 4.4. Prevention of Body Weight and Posture Abnormalities

The obtained research results and available literature indicate the need to monitor the body posture of children from the age of 5, with particular emphasis on overweight and obese people. Many authors also point to the need to introduce fitness classes aimed at the physioprevention of body posture abnormalities in preschool- and school-age children. Although the conducted research does not indicate that overweight and obesity in preschool age influence the occurrence of posture defects, studies conducted by other authors indicate that persistent excess body weight may result in postural defects. The conclusion of this research and literature analysis should be the development of physioprophylactic programs addressed to children aged 5–10 years, aimed at preventing the occurrence of abnormalities in body weight and posture.

The therapeutic program was to determine the possibility of conducting such classes during pre-school hours. However, it did not produce the desired results. As a pilot program, the desired results were confirmed by research on older age groups [36,37], indicating the need to implement physical activity at the reference level set out by WHO (60 min of intense physical exercise daily) into the kindergarten and school program as an element of physioprophylaxis against overweight and obesity, and posture defects.

When summarizing the research conducted, the limitations of the research project were noted. The main problem was the level of participation of children in therapeutic activities. This problem was caused not only by the lack of parental consent or the child’s reluctance, but also by a large number of absences due to illness in the winter and spring periods when classes were held. Another limitation that had the greatest impact on the lack of effects of the therapeutic program was the too-low intensity of classes. The project supplemented the physical activity of preschool children carried out in kindergarten with additional classes once a week, which seems to be an insufficient intervention. The size of the group participating in a single therapeutic session turned out to be a big problem. The classes were held in groups of up to 10–15 people with 2–3 physiotherapists, but the children’s concentration during the classes was low and the group size did not allow for individualized work. At the same time, the training group consisted of both overweight and normal-weight children, which resulted from the educational program implemented in the kindergarten. The examined children attended 11 kindergarten groups and came to classes according to their group membership. It seems that greater effects could be achieved if groups with abnormalities were combined and targeted at specific dysfunctions. However, this was not possible in the presented research.

Thanks to the above research, a project was developed for children of preschool and school age, taking into account the observed limitations.

## 5. Conclusions

The lack of correlation between body weight and postural abnormalities in the studied group at the age of 5–6 may indicate the acquisition of postural defects independent of overweight or obesity persistence.

## Figures and Tables

**Table 1:** Comparison of height, weight, and body composition in both groups.

	Group I n = 51			Group II n = 27			*p* *
	Median	Lower Quartile	Upper Quartile	Median	Lower Quartile	Upper Quartile	
Age [years]	6.0	5.0	6.0	6.0	6.0	6.0	0.007
Height [cm]	116.0	112.0	119.0	120.0	115.0	125.0	0.022
Body weight [kg]	20.6	18.9	22.7	23.5	22.4	27.1	0.000
BMI [kg/m^2^]	15.2	14.7	16.0	16.9	16.2	18.5	0.000
Fat Percentage (FM%) [%]	20.0	18.5	21.9	23.3	21.9	25.7	0.000
Fat-Free Mass (FFM) [kg]	16.6	15.2	18.1	18.4	16.8	20.4	0.001
Overall Muscle Mass (PMM) [kg]	15.7	14.4	17.0	17.4	15.8	19.3	0.001

* U Mann–Whitney test.

**Table 2:** Comparison of body composition in the limbs of both groups.

	Group I n = 51			Group II n = 27			*p* *
	Median	Lower Quartile	Upper Quartile	Median	Lower Quartile	Upper Quartile	
RL FM%	28.9	27.6	30.4	32.1	31.1	33.7	0.000
LL FM%	29.3	27.9	30.4	32.8	31.4	34.1	0.000
*p* **	0.000			0.039			
RL FFM	2.3	2.0	2.6	2.5	2.2	3.0	0.015
LL FFM	2.2	1.9	2.4	2.4	2.1	2.9	0.015
*p* **	0.000			0.003			
RL PMM	2.2	1.9	2.5	2.5	2.2	2.9	0.010
LL PMM	2.2	1.9	2.4	2.4	2.1	2.8	0.010
*p* **	0.000			0.001			

* U Mann–Whitney test; *p* ** the Wilcoxon test.

**Table 3:** Occurrence of posture irregularities.

			Group I [%]	Group II [%]	Chi-Square
Posture		Standing	21.57	22.22	0.947
		Sitting	7.84	11.11	0.631
Head position		In the sagittal plane	33.33	26.92	0.202
		In the frontal plane	3.92	15.38	
Spine	Cervical	Hyperlordosis	13.73	25.93	0.160
		Loss of lordosis	15.69	3.70	
	Thoracic	Hyperkyphosis	13.73	25.93	0.361
		Lordotic stiffening	17.65	11.11	
	Lumbar	Hyperlordosis	47.06	59.26	0.573
		Loss of lordosis	7.84	7.41	
Pelvis			50.98	55.56	0.683
Thoracic cage			23.53	18.52	0.538
Lower limb	Hips:		23.53	18.52	0.610
	Knees		41.18	40.74	0.383
	Feet		52.94	62.96	0.396

**Table 4:** Relationship between anthropometric values and the occurrence of posture irregularities (Spearman’s rank correlations).

		Body Weight	BMI	FM%	FFM	PMM	Lower Limb RIGHT			Lower Limb LEFT		
							RL FM%	RL FFM	RL PMM	LL FM%	LL FFM	LL PMM
Body posture		−0.184	−0.232	−0.125	−0.151	−0.153	−0.052	−0.135	−0.144	−0.111	−0.121	−0.132
Asymmetry		−0.052	−0.07	−0.16	−0.02	−0.032	−0.004	−0.112	−0.099	−0.121	−0.08	−0.069
Sitting body posture		0.178	0.141	−0.00	0.188	0.179	0.050	0.138	0.144	−0.020	0.174	0.183
Spine	Cervical	0.135	0.028	0.059	0.127	0.126	−0.031	0.144	0.136	−0.005	0.159	0.142
	Thoracic	0.133	−0.009	−0.038	0.162	0.159	−0.020	0.132	0.147	0.004	0.139	0.155
	Lumbar	0.000	0.036	0.036	0.003	0.001	0.044	0.022	0.022	0.116	−0.009	-0.001
Pelvis		−0.055	−0.080	−0.012	−0.056	−0.055	0.033	−0.086	−0.084	0.027	−0.062	−0.062
Thoracic cage:		−0.016	−0.060	−0.160	0.027	0.020	−0.038	−0.001	0.014	−0.121	0.014	0.031
Hips:		0.069	0.002	−0.122	0.105	0.101	−0.141	0.094	0.103	−0.116	0.066	0.085
Knees:		−0.356	−0.161	−0.064	−0.361	−0.357	−0.075	−0.297	−0.309	−0.061	−0.302	−0.310
Feet		−0.236	−0.108	−0.052	−0.232	−0.231	−0.049	−0.205	−0.195	−0.074	−0.214	−0.203
		Group I										
Body posture		−0.309	−0.318	−0.244	−0.280	−0.286	−0.008	−0.312	−0.304	−0.153	−0.299	−0.293
Asymmetry		0.029	0.003	−0.137	0.066	0.052	0.098	−0.063	−0.046	−0.004	−0.049	−0.022
Sitting body posture		0.089	0.194	0.060	0.072	0.062	0.129	−0.012	0.000	0.072	0.020	0.037
Spine	Cervical	0.217	0.140	0.081	0.186	0.051	0.201	0.203	0.086	0.184	0.183	0.187
	Thoracic	0.109	−0.030	−0.071	0.126	0.065	0.079	0.111	0.078	0.054	0.088	0.122
	Lumbar	−0.243	−0.121	−0.037	−0.237	0.121	−0.253	−0.240	0.098	−0.259	−0.246	-0.242
Pelvis		−0.206	−0.215	−0.092	−0.206	−0.209	0.036	−0.272	−0.263	−0.007	−0.259	−0.249
Thoracic cage:		−0.036	0.048	−0.125	−0.012	−0.019	0.086	−0.054	−0.039	−0.001	−0.043	−0.018
Hips:		0.104	0.017	−0.035	0.104	0.108	−0.019	0.066	0.079	−0.022	0.071	0.092
Knees:		−0.480	−0.264	−0.033	−0.469	−0.463	0.035	−0.398	−0.414	−0.056	−0.368	−0.392
Feet		−0.404	−0.293	−0.120	−0.386	−0.379	−0.087	−0.321	−0.328	−0.198	−0.292	−0.302
		Group II										
Body posture		0.109	−0.091	0.145	0.109	0.118	−0.091	0.164	0.155	0.018	0.173	0.155
Asymmetry		−0.271	−0.336	−0.394	−0.167	−0.187	−0.251	−0.232	−0.212	−0.468	−0.168	−0.168
Sitting body posture:		0.288	0.000	−0.265	0.356	0.333	−0.235	0.303	0.311	−0.348	0.372	0.379
Spine	Cervical	0.091	−0.127	0.102	0.110	−0.130	0.085	0.090	−0.091	0.174	0.151	0.095
	Thoracic:	0.252	0.016	−0.080	0.315	−0.316	0.263	0.259	−0.212	0.311	0.310	0.303
	Lumbar	0.411	0.201	0.144	0.404	−0.228	0.453	0.444	0.089	0.365	0.366	0.401
Pelvis		0.201	0.110	0.148	0.215	0.220	−0.057	0.201	0.192	0.043	0.249	0.225
Thoracic cage:		0.145	−0.064	−0.195	0.161	0.161	−0.199	0.153	0.161	−0.287	0.194	0.200
Hips:		0.104	0.049	−0.337	0.147	0.122	−0.282	0.184	0.196	−0.251	0.117	0.141
Knees:		−0.209	−0.051	−0.077	−0.209	−0.207	−0.230	−0.114	−0.128	−0.061	−0.191	−0.185
Feet		−0.123	0.049	−0.163	−0.094	−0.094	−0.276	−0.049	−0.020	−0.158	−0.109	−0.074

## Data Availability

The research results are available on request from the corresponding author.
